# *SLC6A3*, *HTR2C* and *HTR6* Gene Polymorphisms and the Risk of Haloperidol-Induced Parkinsonism

**DOI:** 10.3390/biomedicines10123237

**Published:** 2022-12-13

**Authors:** Gordana Nedic Erjavec, Mirko Grubor, Maja Zivkovic, Nada Bozina, Marina Sagud, Matea Nikolac Perkovic, Alma Mihaljevic-Peles, Nela Pivac, Dubravka Svob Strac

**Affiliations:** 1 Laboratory for Molecular Neuropsychiatry, Division of Molecular Medicine, Rudjer Boskovic Institute, 10 000 Zagreb, Croatia; 2 Faculty of Pharmacy and Biochemistry, University of Zagreb, 10 000 Zagreb, Croatia; 3 Department of Psychiatry, University Hospital Centre Zagreb, 10 000 Zagreb, Croatia; 4 School of Medicine, University of Zagreb, 10 000 Zagreb, Croatia

**Keywords:** schizophrenia, haloperidol, parkinsonism, serotonin receptors, dopamine transporter, gene polymorphisms, genotype, haplotype, interaction

## Abstract

Antipsychotic-induced parkinsonism (AIP) is the most common type of extrapyramidal side effect (EPS), caused by the blockage of dopamine receptors. Since dopamine availability might influence the AIP risk, the dopamine transporter (DAT) and serotonin receptors (5-HTRs), which modulate the dopamine release, may be also involved in the AIP development. As some of the individual differences in the susceptibility to AIP might be due to the genetic background, this study aimed to examine the associations of *SLC6A3, HTR2C* and *HTR6* gene polymorphisms with AIP in haloperidol-treated schizophrenia patients. The Extrapyramidal Symptom Rating Scale (ESRS) was used to evaluate AIP as a separate entity. Genotyping was performed using a PCR, following the extraction of blood DNA. The results revealed significant associations between *HTR6* rs1805054 polymorphism and haloperidol-induced tremor and rigidity. Additionally, the findings indicated a combined effect of *HTR6* T and *SLC6A3* 9R alleles on AIP, with their combination associated with significantly lower scores of ESRS subscale II for parkinsonism, ESRS-based tremor or hyperkinesia and ESRS subscales VI and VIII. These genetic predictors of AIP could be helpful in better understanding its pathophysiology, recognizing the individuals at risk of developing AIP and offering personalized therapeutic strategies for the patients suffering from this EPS.

## 1. Introduction

Drug-induced movement disorders are neurological adverse effects caused by the drugs that block dopamine receptors, with antipsychotics being the most common cause. Antipsychotic-induced parkinsonism (AIP) is the most common type of extrapyramidal side effect (EPS) [1]. Clinically, it is characterized by akinesia, tremor, bradykinesia, rigidity and postural instability, and usually develops within a few days or weeks after the start of antipsychotic treatment [2]. The average annual incidence rate of AIP was estimated to be 3.3 per 100,000 people [3]. AIP considerably decreases the patient’s daily functioning and quality of life, and it is one of the major reasons for the poor adherence of schizophrenia patients to antipsychotic therapy, which increases the likelihood of disease recurrence and worse prognosis [4,5,6]. Moreover, it has been demonstrated as a risk factor for the development of later tardive dyskinesia [7,8,9].

All antipsychotics have a potential of developing AIP, ranging from low to high [6]. However, up to 40–50% of patients using typical antipsychotics develop AIP [10], with haloperidol demonstrating the highest risk [6]. Specifically, according to the theory of do-pamine receptor blockade [11], the symptoms of parkinsonism emerge when more than 80% of striatal dopamine 2 receptors (DRD2) are blocked [12,13,14]. Therefore, a higher affinity of typical antipsychotics for DRD2, in comparison to atypical antipsychotics, may account for their increased liability for AIP [15,16]. However, since the risk of developing AIP with some newer atypical antipsychotics remains high [11,17], beyond the effect of the drug on dopamine neurons, other possible mechanisms should be considered [6,18]. Research suggested that the pathophysiology of AIP involves complex interactions of multiple neurotransmitter systems (dopamine, serotonin, norepinephrine, acetylcholine, etc.) that are influenced by non-modifiable risk factors such as gender, age, ethnicity and genetic predisposition, as well as by the factors which could be adjusted, including the type, dose and treatment duration of the antipsychotic drug, concomitant diseases, etc. [3,6,19]. Since some of the individual differences in the susceptibility to AIP might be due to the genetic background, various studies were carried out to search for different candidate genes involved in the pharmacokinetic and pharmacodynamic pathways, which could represent the predictors of the AIP development [20,21,22,23].

Given the proposed pharmacological mechanism of action for antipsychotics, variants of the genes related to the dopaminergic system are of most interest in the AIP research. The lower dopamine availability in the synaptic cleft might facilitate the antipsychotic blockade of DRD2, and thus influence the AIP risk [24]. The dopamine transporter (DAT) plays an important role in dopamine availability and dopaminergic neurotransmission by mediating the active reuptake of dopamine from the synaptic cleft into neurons. There is even a hypothesis that, other than DRD2, DAT might be a main target of antipsychotic drugs, suggesting that DAT blockers may be used as an adjunct treatment to reverse antipsychotic treatment failure [25]. Since the serotonergic system interacts with the dopaminergic function, and serotonin receptors (5-HTRs) modulate the dopamine release [26], they may be also involved in the development of AIP [26,27]. Many antipsychotic drugs, in particularly atypical antipsychotics, have a high affinity for 5-HT2AR and 5-HT2CR. The antagonism of 5-HT2AR/2CR has been shown to alleviate antipsychotic-induced EPS by relieving the inhibition of nigral dopaminergic activity and striatal dopamine release [28,29,30]. Specifically, the 5-HT2CR possesses a unique ability to tonically regulate dopamine release from the nigrostriatal pathway, and might play an important role in AIP [31]. Additionally, there are studies implicating that 5-HT6R antagonists have a protective effect against the development of motor disorders induced by haloperidol and other antipsychotics [30,32].

There are already reports about the role of DAT and 5-HTR gene polymorphisms in the development of EPS [20,33,34,35,36]. However, the obtained results have been limited and are often contradictory. Moreover, none of these studies investigated the association between these genetic variations and AIP as a separate entity. Although the pathophysiology of different EPS is still not clear, each EPS type is characterized with specific features and different neuroanatomical patterns with potentially variant genetic vulnerability [37]. In addition, most of these studies evaluated EPS using the Simpson–Angus Scale (SAS). Although the SAS was developed to measure AIP and it was shown to be a valid, reliable and easy-to-use instrument [38], questions have arisen on whether this scale properly evaluates the different aspects of parkinsonism [39]. Therefore, the aim of this study was to investigate the potential association of selected polymorphisms, located in the *HTR2C*, *HTR6* and *SLC6A3* genes, coding for 5-HT2CR, 5-HT6R and DAT, respectively, with the haloperidol-induced parkinsonism in schizophrenia patients, by using another well-validated rating scale, namely, the Extrapyramidal Symptom Rating Scale (ESRS) [40,41]. Genetic predictors of AIP could be helpful in better understanding its pathophysiology, predicting the individuals at higher risk of developing AIP as well as in offering personalized therapeutic strategies for the patients suffering from this particular EPS.

## 2. Materials and Methods

### 2.1. Participants and Clinical Evaluation

The study included 229 male patients with schizophrenia whose socio-demographic and clinical characteristics were published previously [20]. All participants were Caucasians of Croatian origin. Patients were recruited during their admission to the Psychiatric Hospital Popovaca and the Department of Psychiatry, University Hospital Centre Zagreb, Croatia, due to acute schizophrenia exacerbation. Schizophrenia was diagnosed by psychiatrists according to the Diagnostic and Statistical Manual of Mental Disorders, Fourth Edition (DSM-IV) criteria [42], and the evaluation of the severity of schizophrenia symptoms was based on the Positive and Negative Syndrome Scale (PANSS) [43]. Patients were treated for two weeks with antipsychotic drug haloperidol (15 mg/day, orally or intramuscularly). They received no concomitant medication during the study except adjuvant diazepam therapy (40 mg daily), which was introduced for the treatment of agitation, insomnia and anxiety. All of the patients had no previous antipsychotic therapy for at least 48 h. In total, 205 out of 229 patients with schizophrenia used antipsychotic medication previously (89.52%), usually a combination of typical and atypical antipsychotics (69.00%). However, most of the subjects had not taken antipsychotics for several months, and some of them (10.48%) were drug naïve. Patients with serious somatic illnesses, neurologic disorders and a history of drug use during the previous 6 months were not included in the study. Additionally, only patients who signed the informed consent were enrolled. The study was carried out in accordance with the Declaration of Helsinki [44], and approved by the ethics committees of the Psychiatric Hospital Popovaca and the University Hospital Centre Zagreb, Croatia. Haloperidol-induced parkinsonism was assessed in patients with schizophrenia using the Extrapyramidal Symptom Rating Scale (ESRS). The ESRS consists of five sections: a patient questionnaire and four subsections based on clinical observation and examination. The patient questionnaire rates the subjective experience of EPS in patients during the preceding week, whereas four subsections assess four types of drug-induced movement disorders: parkinsonism, akathisia, dystonia and tardive dyskinesia [40]. The score for parkinsonism ranges from 0 to 96 as a sum of 6 items of the ESRS subscale II: tremor (score 0–48), gait and posture (score 0–6), postural stability (score 0–6), rigidity (score 0–24), expressive automatic movements (score 0–6) and bradykinesia (score 0–6) [41]. In this study, AIP was evaluated according to scores of ESRS subscale II items, subscores hypokinesia (sum of scores for the items gait and posture, rigidity, expressive automatic movements and bradykinesia) and hyperkinesia (score for tremor), the clinical global impression of severity (CGI-S) of parkinsonism and the stage of parkinsonism [45], as instructed by Chouinard and Margolese [41].

### 2.2. Sampling and Genotyping

Samples of subjects’ venous blood (4 mL) were collected using a plastic syringe containing 1 mL acid citrate dextrose as an anticoagulant and were used for isolation of genomic DNA conducted by a standard salting-out method [46]. Single nucleotide polymorphisms (SNPs) coding for 5-HTRs, *HTR2C* rs3813929, *HTR2C* rs518147 and *HTR6* rs1805054, as well as the variable number of tandem repeat (VNTR) polymorphism, 3′UTR VNTR in *SLC6A3* gene coding for DAT, were analyzed. Genotyping for *HTR2C* rs3813929 and rs518147 polymorphisms, as well as for *HTR6* rs1805054 polymorphism, was performed using the ABI Prism 7000 Sequencing Detection System apparatus (Applied Biosystems, Foster City, CA, USA), according to the procedures described by Applied Biosystems. The primers and probes were purchased from Applied Biosystems as TaqMan^®^ SNP Genotyping Assays (C__27488117_10 for rs3813929, C___2308053_10 for rs518147 and C___1264819_10 for rs1805054). The 6 alleles of the *SLC6A3* 3′UTR VNTR polymorphism consisting of 6, 7, 8, 9, 10 or 11 copies of the 40-base-pair repeat sequence were analyzed using a GeneAmp PCR System 9700 (Applied Biosystems, Foster City, CA, USA) by polymerase chain reaction and primers 5′-TGTGGTGTAGGGAACGGCCTGAG-3′ and 5′-CTTCCTGGAGGTCACGGCTCAAGG-3′ with reaction conditions as follows: 4 min at 94 °C, 35 cycles of 94 °C for 45 s, 70 °C for 2 min and 72 °C for 30 s and final extension at 72 °C for 10 min.

### 2.3. Data Analyses

The results were expressed as median and 25th (Q1) and 75th (Q3) percentiles and evaluated using GraphPad Prism version 4.00 for Windows (GraphPad Software, Inc., San Diego, CA, USA). Normality of the distribution was assessed with the Kolmogorov–Smirnov test. Since the data were not normally distributed, they were compared by non-parametric tests (Mann–Whitney U-test for two groups and Kruskal–Wallis H test with post-hoc Dunn’s test for more than two groups). The deviation from the Hardy–Weinberg equilibrium (HWE) was tested using the χ^2^-tests. Due to the multiple testing (four polymorphisms), the Bonferroni correction was applied, and the p value was set to 0.012. Haploview 4.2 software (Broad Institute of Harvard and MIT, Cambridge, MA, USA) [47] was used to test the linkage disequilibrium between the rs3813929 and rs518147 polymorphisms of the *HTR2C* gene. The estimation of haplotype pairs for each subject was made using the gPLINK 2.050 software tool [48]. A priori determination of sample size and post hoc computation of the achieved power were conducted by G*Power 3.1 Software [49]. For the Kruskal–Wallis test with α = 0.012, power = 0.80 and medium effect size (0.25), the total desired sample size was 222. For the Mann–Whitney test with α = 0.012, power = 0.80 and medium effect size = 0.50), the total desired sample size was 200. As the actual total sample size was 229, the power analysis confirmed the appropriate sample size, and thus the statistical power of the study.

## 3. Results

All the clinical and demographic data for 229 participants enrolled in this study were previously described in detail [20]. To summarize, the subjects’ age expressed as median (Q1; Q3) was 35 (29; 45). Most of them (89.5%) previously received antipsychotic therapy, usually as a combination of typical and atypical antipsychotics (69.0%). All subjects were admitted to the hospital due to the acute exacerbation of schizophrenia which could be illustrated by high baseline positive (36 (32; 39), negative (35 (31; 38)), general psychopathology (62 (56; 66) and total (132 (123; 140)) Positive and Negative Syndrome Scale (PANSS) scores. A total of 66.81% of the schizophrenia patients reported some kind of EPS, which usually developed on the 5th day of haloperidol monotherapy. AIP examination scores, evaluated by the Extrapyramidal Symptom Rating Scale (ESRS) and indicating overall mild parkinsonism, are shown in Table 1. The ESRS detected some symptoms of parkinsonism in ~58% of the haloperidol-treated subjects enrolled in the study [20].

Both *HTR2C* polymorphisms were successfully determined in 229 schizophrenia patients, whereas 219 patients were successfully genotyped for *HTR6* polymorphism and 216 patients for *SLC6A3* polymorphism. Out of 216 patients with obtained *SLC6A3* genotypes, 7 subjects were excluded because they were homozygous for rare alleles (6, 7 and 11 copies). The genotype distributions for all studied polymorphisms did not deviate from the Hardy–Weinberg equilibrium. A haplotype analysis conducted by Haploview software revealed that the two investigated *HTR2C* polymorphisms (rs3813929 and rs518147) were in linkage disequilibrium (D’ = 0.85). The frequencies of the four detected haplotypes were 68% for CG, 18% for TC, 13% for CC and 1% for TG haplotype. Due to its negligible frequency, the TG haplotype was excluded from further analyses.

When subjects were divided according to the *HTR2C* rs3813929 (Supplementary Table S1) or *HTR2C* rs518147 (Supplementary Table S2) genotypes, no differences in the scores of the measured ESRS subscales or items between the groups were found. There was also no association between the ESRS-based indicators of parkinsonism and *HTR2C* haplotypes (Supplementary Table S3). However, when we compared the ESRS-based AIP indicators between the carriers of certain *HTR2C* haplotypes (Table 2), we detected a nominally significant (*p* = 0.036) association (rejected due to the Bonferroni correction) of the *HTR2C* CC haplotype with the severity of parkinsonism, evaluated with the ESRS subscale VI.

**Figure 1:**
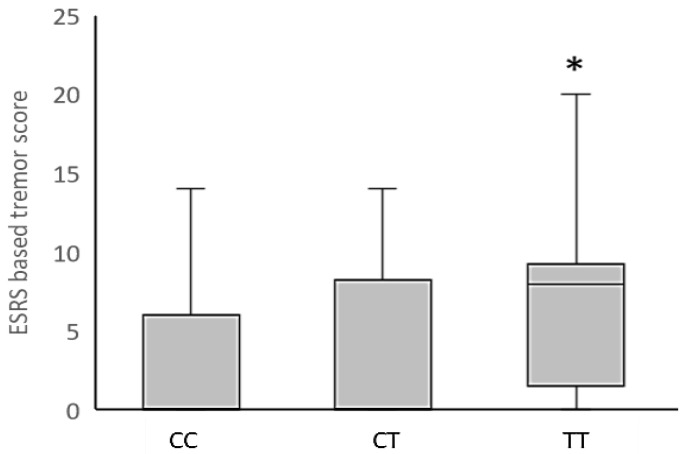
Distribution of ESRS-based tremor/hyperkinesia scores in haloperidol-treated schizophrenia patients with different *HTR6* rs1805054 genotypes. Data are expressed as median (Q1; Q3). * *p* = 0.011 vs. CC genotype carriers, Dunn’s post-hoc test following Kruskal–Wallis analysis.

In addition, the Kruskal–Wallis analysis revealed a significant association between the *HTR6* rs1805054 genotype and haloperidol-induced tremor in schizophrenia patients (Figure 1), with the increased ESRS-based tremor/hyperkinesia scores in carriers of the *HTR6* TT genotype (*p* = 0.011, Dunn’s post-hoc test), compared to CC genotype carriers, being the main contributor to this significant finding. Nominally significant associations of the *HTR6* rs1805054 genotype with ESRS subscale II for parkinsonism (H = 6.06, *p* = 0.048) and ESRS-based rigidity (H = 6.90; *p* = 0.032) scores were rejected due to the Bonferroni correction (Supplementary Table S4). No significant associations between the *HTR6* rs1805054 polymorphism and scores on the other applied ESRS-based AIP examination instruments were found (Supplementary Table S4).

When investigating whether the ESRS-based parkinsonism indicators were different in carriers of particular *HTR6* rs1805054 alleles, we observed several nominally significant associations with the scores on different ESRS subscales and items for AIP (Table 3). However, the only significant finding that survived the Bonferroni correction was higher ESRS-based rigidity scores in *HTR6* T allele carriers (*p* = 0.010) in comparison to the *HTR6* CC homozygotes (Table 3).

Moreover, no associations between *SLC6A3* 3′UTR VNTR genotypes and scores at any of the included ESRS subscales and items for AIP were found (Supplementary Table S5). In haloperidol-treated schizophrenia patients carrying the *SLC6A3* 3′UTR VNTR alleles with 9 or 10 repeats, only nominally significant findings of the higher scores at ESRS subscales VI and VIII in the carriers of 9 repeats (9R) allele were determined (Table 4).

Although most of the initially observed significant results have not survived the Bonferroni correction for multiple comparisons, several associations of the studied serotonergic and dopaminergic polymorphisms with the measured ESRS-based indicators of haloperidol-induced parkinsonism were detected. In order to check if their combination could exert a stronger effect on AIP, further statistical analyses included gene variants shown to be associated with the scores on most ESRS subscales and items.

Therefore, the haloperidol-treated schizophrenia patients were grouped according to the combined presence of *HTR6* T and *SLC6A3* 9R alleles, which were identified as risk alleles for AIP. For that purpose, the ESRS-based AIP indicators were compared between three groups: the carriers of both *HTR6* T and *SLC6A3* 9R risk alleles; the carriers of either *HTR6* T or *SLC6A3* 9R risk allele; and the carriers of neither *HTR6* T nor *SLC6A3* 9R risk alleles (i.e., carriers of *HTR6* C and S*LC6A3* 10R alleles).

The results indicated that haloperidol-treated schizophrenia patients carrying neither *HTR6* T nor *SLC6A3* 9R AIP-risk alleles (i.e., carriers of *HTR6* C and *SLC6A3* 10R alleles) have significantly lower scores at ESRS subscale II for parkinsonism, ESRS-based tremor or hyperkinesia and ESRS subscales VI and VIII, in comparison to the carriers of either *HTR6* T or *SLC6A3* 9R risk allele or carriers of both *HTR6* T and S*LC6A3* 9R risk alleles (Table 5). The same trend was visible also for the ESRS-based rigidity score; however, due to the Bonferroni correction, this result was only nominally significant (Table 5). Therefore, these results suggested that schizophrenia patients, carriers of either single or both risk alleles, have higher scores for ESRS-based indicators of AIP, indicating more severe symptoms of haloperidol-induced parkinsonism.

## 4. Discussion

This study investigated the associations of *HTR2C* rs3813929 and rs518147, *HTR6* rs1805054 and *SLC6A3* 3′UTR VNTR polymorphisms with haloperidol-induced parkinsonism, evaluated using the ESRS, in 229 male patients with schizophrenia. The present study represents an extension of our previous research, which demonstrated significant associations of acute haloperidol-induced EPS with *SLC6A3* 3′UTR VNTR and *COMT* Val158Met polymorphisms [33], as well as a significant association of haloperidol-induced akathisia with the *HTR1B* rs13212041 gene polymorphism [20].

In contrast to our earlier results [33], in the present study we have determined only nominally higher scores at ESRS subscales VI and VIII for AIP in the haloperidol-treated schizophrenia patients carrying the 9 repeats (9R) allele of *SLC6A3* 3′UTR VNTR polymorphism. The association of the *SLC6A3* 3′UTR VNTR with antipsychotic-induced EPS has been previously investigated [50,51,52,53]; however, only few studies observed a significant association [33,35].

The *SLC6A3* 3′UTR VNTR is a 40-base-pair (bp) variable number of tandem repeat (VNTR) polymorphism located in the exon 15 within the 3-untranslated region of the *SLC6A3* gene coding for a dopamine transporter (DAT). Variable numbers of the 40-bp repeat range from 3 to 11 copies, with the 9-repeat (9R) and 10-repeat (10R) representing the two most common alleles of this polymorphism [54]. Although preclinical studies indicated that the *SLC6A3* 3′UTR VNTR polymorphism influences gene expression and DAT protein levels, contradictory results have been observed for the gene expression of each allele [55,56,57,58]. Inconsistent findings were also reported regarding the influence of different *SLC6A3* 3′UTR VNTR alleles on DAT binding [59,60,61,62]. In addition, Mill et al. (2005) suggested that this polymorphism might not have a direct effect on DAT expression, but rather via linkage disequilibrium with other functional polymorphisms [63].

DAT plays an important role in the regulation of dopamine levels through dopamine reuptake from the synapse into neuronal cells. The patients who develop AIP may have lower dopamine availability in the synaptic cleft, which could facilitate the DRD2 blockade with antipsychotic drugs, and thus influence the risk of AIP development [24]. Moreover, some authors even suggested that DAT could be a main target of antipsychotic drugs [25].

Serotonin receptors (5-HTRs) can modulate the dopamine release as well [64], and serotonin inhibition of dopamine function may also contribute to AIP [26,27]. Among the various 5-HTRs, the 5-HT2AR and 5-HT2CR have been the most extensively studied, since various antipsychotic drugs, especially atypical antipsychotics, have a high affinity for these receptors. The 5-HT2CR are expressed in basal ganglia, brain regions important for movement disorders [65,66], and exert inhibitory action on the dopaminergic system [67,68]. Since 5-HT2CR tonically regulates dopamine release from the nigrostriatal pathway [31], a protective mechanism against EPS might be achieved via 5-HT2CR antagonism that relieves the inhibition of nigral dopaminergic activity and striatal dopamine release [28,29,30,69].

The *HTR2C* rs3813929 polymorphism, also known as −759C/T, and *HTR2C* rs518147 polymorphism, also known as −697G/C, are located in the promoter region of the *HTR2C* gene [70]. Regarding *HTR2C* rs3813929 variants, the −759C allele showed lower transcriptional activity in comparison to the −759T allele [71], whereas the −697C allele of *HTR2C* rs518147 polymorphism has been associated with lower promoter activity compared to the −697G variant [72]. Several studies reported associations of antipsychotic-induced acute EPS, including parkinsonism, with polymorphisms located in the *HTR2C* gene [36,73,74]. In contrast, our previous findings suggested no significant associations between *HTR2C* polymorphisms and haloperidol-induced EPS [20], whereas the present study revealed only a nominally significant association of the *HTR2C* CC haplotype with the severity of parkinsonism, evaluated with the ESRS subscale VI.

Both 5-HT2CR and 5-HT6R were shown to mediate the 5-HT-induced activation of the striatal acetylcholine neurons, implicated in the antipsychotic-induced EPS [75]. Therefore, the observed decrease in the incidence and severity of EPS in the presence of 5-HT6R antagonists, might reflect a decrease in the activation of striatal acetylcholine neurons [30,32]. Specifically, the blockade of 5-HT6R has been shown to attenuate the haloperidol-induced motor disorders [32]. The *HTR6* rs1805054 polymorphism, also called C267T, is a synonymous polymorphism which has been revealed in exon 1, corresponding to Tyr89 [76]. It has been predominantly investigated in association with schizophrenia [77,78,79], and its response to antipsychotic drugs [80,81]. This is not surprising, since antipsychotics such as clozapine, quetiapine and olanzapine, but also tricyclic antidepressants, as well as tryptamine and ergoline derivatives, possess an affinity for the 5-HT6R [76,82].

In contrast to our previous findings [20], present data revealed significant association between *HTR6* rs1805054 polymorphism and haloperidol-induced tremor and rigidity. These contradictive results might be due to the fact that in the previous study we examined the association of *HTR6* rs1805054 polymorphism with acute EPS [20], whereas in this study, we investigated the association between this genetic variation and AIP as a separate entity. Specifically, it has been suggested that each EPS type is characterized by specific features and different neuroanatomical patterns with potentially variant genetic vulnerability, and therefore should be assessed separately [37].

In addition, we also detected a possible combined effect of *HTR6* T and *SLC6A3* 9R alleles on AIP, with their particular combination associated with significantly lower scores of ESRS subscale II for parkinsonism, ESRS-based tremor or hyperkinesia and ESRS subscales VI and VIII. These results suggested that schizophrenia patients, carriers of either single or both *HTR6/SLC6A3* risk alleles, have higher scores for ESRS-based indicators of AIP, i.e., more severe EPS. Our earlier study [33] did not observe a significant interaction of *SLC6A3* 9/10 alleles with alleles of *DRD2* or *COMT* Val/Met polymorphisms in the development of acute EPS. However, to the best of our knowledge, this is the first work that shows that the combination of *HTR6* and *SLC6A3* risk alleles is associated with the development of haloperidol-induced parkinsonism.

Unlike most research, which evaluated the EPS using the SAS, we have used the ESRS [40,41], and selected its particular subsections in order to specifically evaluate various indicators of parkinsonism in haloperidol-treated schizophrenia patients. Although the SAS became the standard measurement instrument for AIP due to its validity, reliability and easy usage [38], some concerns appeared on whether this scale properly evaluates the different aspects of parkinsonism. Specifically, the SAS lacks any measure of bradykinesia, but uses some unreliable measures such as glabella reflex and pooled saliva in the mouth, and it is overly reliant on rigidity [39].

Our study with an appropriate sample size and statistical power suggested some potential pharmacogenetic predictors of haloperidol-induced parkinsonism, and hopefully improved the current understanding of AIP pathophysiology. This is important since AIP negatively affects the patient’s everyday functions and life quality and often results in reduced patient compliance or even discontinuation of the therapy, which are closely linked to the exacerbation of symptoms and disease relapse [4,5,6]. In addition, patients who have experienced AIP also show a predisposition to develop tardive dyskinesia [7,8,9,83,84].

The schizophrenia patients enrolled in our study received haloperidol, a highly effective and widely prescribed classical antipsychotic [85,86], which demonstrates the highest risk of AIP [6,87] due to the “tight” blockade of the DRD2 in the nigrostriatal pathway [11,12,14,15,88]. Other typical antipsychotics also have increased liability for AIP [10,15,16] due to a higher affinity for DRD2, when compared to atypical antipsychotics. However, all antipsychotics have some potential of developing AIP [6], and the risk of developing EPS with some newer atypical antipsychotics remains high [11,17]. Therefore, the management of schizophrenia remains a substantial clinical challenge. In addition, AIP is not limited to antipsychotic medication, as it may be also caused by some gastrointestinal motility drugs, calcium channel blockers, antidepressants and antiepileptic drugs [2,89,90,91]. This should be taken into account since, faced with a patient that does not respond sufficiently to antipsychotics or cannot tolerate standard antipsychotic therapy, physicians often need to prescribe the combination therapy [92]. Therefore, further studies should test a wider range of antipsychotics and other drugs for the association between the investigated polymorphisms and AIP.

Although haloperidol monotherapy allowed us to exclude possible interactions between different antipsychotic drugs, we cannot completely rule out a possible effect of concomitant diazepam on haloperidol-induced parkinsonism [93,94]. Moreover, we have not determined the patients’ plasma concentrations of haloperidol or CYP2D6 genotypes that could influence the metabolic capacity of the CYP2D6 enzyme [95]. It is known that individuals carrying the genotypes resulting in poor CYP2D6 metabolism could have higher plasma levels of antipsychotics, and therefore an increased risk of AIP development [96]. Since lower dopamine availability in the synaptic cleft might facilitate the antipsychotic blockade of DRD2 and thus influence the AIP risk, both the *CYP2D6* variants that could influence the capacity of haloperidol metabolism, as well as the variants of the *SLC6A3, HTR2C* and *HTR6* genes, that could modulate the dopamine release, might contribute to the potential of haloperidol-induced parkinsonism development. Therefore, future studies should analyze the possible interaction between *CYP2D6* gene variants and the variants of the *SLC6A3, HTR2C* and *HTR6* genes on the risk for haloperidol-induced parkinsonism.

In addition, our study enrolled only male Caucasian schizophrenia patients of Croatian origin. Since some gender differences in the treatment response and antipsychotic side effects [97,98], as well as gender and ethnic differences in the allele frequency distributions [99,100], have been reported, future studies investigating genetic associations with AIP should include both male and female schizophrenia patients of different ethnicities.

To summarize, our study on AIP has several strengths and limitations. We have investigated the associations of individual *SLC6A3, HTR2C* and *HTR6* gene polymorphisms; *HTR2C* haplotypes; as well as the combination of *HTR6* and *SLC6A3* risk alleles with haloperidol-induced parkinsonism in schizophrenia patients. Unlike most research, which evaluated the EPS using the SAS, we have used the ESRS, and selected its particular subsections in order to specifically evaluate various indicators of parkinsonism as a separate entity. Moreover, our study had an appropriate sample size and statistical power and involved an ethnically homogenous group of male middle-aged Caucasian schizophrenia patients of Croatian origin in the acute episode of illness. Another advantage of our study is that the patients were treated with haloperidol monotherapy, which allowed us to exclude possible interactions between different antipsychotic drugs. However, there are some limitations to our study. We have not determined the patients’ plasma concentrations of haloperidol or *CYP2D6* genotypes that could influence the capacity of the CYP2D6 enzyme to metabolize haloperidol. In addition, although haloperidol-treated patients received no concomitant medication except diazepam, we cannot completely rule out a possible effect of diazepam on haloperidol-induced parkinsonism. The lack of female patients, patients of other ethnic origins as well as healthy control subjects in our study also limits its interpretation. Furthermore, some relevant patient information, such as the duration of untreated psychosis and the total duration of the illness, was not taken into account. Another study limitation is a lack of replication of our findings in an independent sample.

Based on accumulating data, including findings of the present study, it is obvious that AIP could not be explained by a simple interaction between antipsychotic drug and DRD2 or by the presence of a single genetic variant with a major effect, but rather by a complex relationship between various genetic variants created by epistasis [101]. Specifically, AIP probably occurs due to the interaction of multiple polymorphisms, with discrete effects and low penetrance, located either within the same or in different genes involved in drug metabolism and transport, as well as in several neurotransmitter systems, and interacting with a variety of non-genetic factors, such as age, type and dose of antipsychotic drug, comorbid diseases, etc.

## 5. Conclusions

The a priori identification of schizophrenia patients with a genetic susceptibility to develop AIP would be useful for guiding clinicians in their choice of antipsychotic drugs and other alternative treatments, as well as for adjusting their dosage, and should limit misdiagnosed EPS. For the patient, this could mean a reduction in the incidence of AIP and better compliance, resulting in a lower risk of medication discontinuation and treatment non-adherence, as strong predictors of relapse and rehospitalization. Since the efficacy of a pharmacological treatment cannot be interpreted independently from its adverse effects profile, understanding and predicting liability to AIP may represent a useful strategy to improve the treatment response, as well as prognosis in schizophrenia patients.

Therefore, further studies should be conducted to test a wider range of antipsychotics and other drugs for the association of the *SLC6A3, HTR2C* and *HTR6* polymorphisms, as well as *CYP2D6* gene variants with AIP, in both male and female schizophrenia patients of different ethnicities. Moreover, it is essential to continue the research of complex interactions between candidate gene variants, in order to increase their predictive capacity for AIP. If confirmed, the *HTR6* rs1805054 polymorphism, alone and especially in combination with the *SLC6A3* 3′UTR VNTR polymorphism, could serve as a pharmacogenetic predictor of AIP, which would be potentially helpful in tailoring personalized therapeutic strategies for the patients at increased risk of developing drug-induced parkinsonism or already suffering from this EPS.

## Figures and Tables

**Table 1:** Scores of individual ESRS subscales and items evaluating AIP in haloperidol-treated schizophrenia patients.

Parkinsonism Examination Instrument	Score Median (Q1; Q3)
ESRS subscale II for parkinsonism	5 (0; 17)
*Expressive automatic movements (facial mask/speech)*	0 (0; 2)
*Bradykinesia*	0 (0; 2)
*Rigidity*	0 (0; 6)
*Gait and posture*	0 (0; 2)
*Tremor*	0 (0; 6)
*Postural stability*	0 (0; 1)
ESRS subscale VI (CGI-S of parkinsonism)	2 (0; 4)
ESRS subscale VIII (stage of parkinsonism)	2 (0; 3)
ESRS subscore hyperkinesia	0 (0; 6)
ESRS subscore hypokinesia	0 (0; 12)

The individual items of ESRS subscale II for parkinsonism are shown in italics.

**Table 2:** ESRS-based AIP scores in haloperidol-treated schizophrenia patients with different *HTR2C* rs3813929-rs518147 haplotypes.

Parkinsonism Examination Instrument	ESRS-Based AIP Scores in Different *HTR2C* Haplotype Carriers					
	Non-TC Haplotypes	TC Haplotype	Non-CG Haplotypes	CG Haplotype	Non-CC Haplotypes	CC Haplotype
ESRS subscale II for parkinsonism	8 (0; 19)	5 (0; 17)	3 (0; 19)	9 (0; 18)	7 (0; 18)	0 (0; 12)
	U = 2879.00; *p* = 0.886		U = 4616.50; *p* = 0.391		U = 2015.50; *p* = 0.311	
Expressive automatic movements	0 (0; 2)	0 (0; 1)	0 (0; 1)	0 (0; 2)	0 (0; 2)	0 (0; 1)
	U = 2877.50; *p* = 0.873		U = 4479.50: *p* = 0.601		U = 2154.00; *p* = 0.592	
Bradykinesia	0 (0; 2)	0 (0; 2)	0 (0; 1)	0 (0; 2)	0 (0; 2)	0 (0; 1)
	U = 2924.50; *p* = 0.994		U = 4545.50; *p* = 0.483		U = 2041.00; *p* = 0.332	
Rigidity	0 (0; 6)	0 (0; 6)	0 (0; 6)	0 (0; 6)	0 (0; 6)	0 (0;6)
	U = 2864.50; *p* = 0.838		U = 447.50; *p* = 0.669		U = 2199.00; *p* = 0.723	
Gait and posture	0 (0; 2)	0 (0; 2)	0 (0; 2)	0 (0; 2)	0 (0; 2)	0 (0;2)
	U = 2984.00; *p* = 0.829		U = 4475.50; *p* = 0.613		U = 2051.50; *p* = 0.349	
Tremor/ESRS subscore hyperkinesia	0 (0; 8)	0 (0; 6)	0 (0; 6)	0 (0; 8)	0 (0; 8)	0 (0; 7)
	U = 2841.00; *p* = 0.765		U = 4516.50; *p* = 0.513		U = 2153.50; *p* = 0.577	
Postural stability	0 (0;1)	0 (0; 0)	0 (0; 0)	0 (0; 1)	0 (0; 1)	0 (0; 0)
	U = 2830.00; *p* = 0.707		U = 4517.50; *p* = 0.467		U = 2163.50; *p* = 0.567	
ESRS subscale VI (CGI-S of parkinsonism)	3 (0; 4)	3 (0; 4)	1 (0; 4)	3 (0; 4)	3 (0; 4)	0 (0; 3)
	U = 3146.50; *p* = 0.459		U = 4637.00; *p* = 0.359		U = 1727.50; *p* = *0.036*	
SRS subscale VIII (stage of parkinsonism)	2 (0; 3)	2 (0; 3)	1 (0; 3)	2 (0; 3)	2 (0; 3)	0 (0; 3)
	U = 2923.50; *p* = 0.997		U = 4710,50; *p* = 0.257		U = 1877.00; *p* = 0.120	
ESRS subscore hypokinesia	1 (0; 12)	3 (0; 9)	0 (0; 11)	2 (0; 12)	2 (0; 12)	2 (0; 12)
	U = 2950.00; *p* = 0.926		U = 4469.50; *p* = 0.638		U = 2091.50; *p* = 0.453	

Data are expressed as median (Q1; Q3) and compared with Mann–Whitney U test. Non-TC haplotypes = carriers of CG and CC haplotypes. Non-CG haplotypes = carriers of TC and CC haplotypes. Non-CC haplotypes = carriers of TC and CG haplotypes. A nominally significant result, rejected after the Bonferonni correction, is shown in italics.

**Table 3:** ESRS-based AIP scores in haloperidol-treated schizophrenia patients with different *HTR6* rs1805054 alleles.

Parkinsonism Examination Instrument	ESRS-Based AIP Scores in Different *HTR6* Allele Carriers			
	TT Homozygotes	C Allele Carriers	CC Homozygotes	T Allele Carriers
ESRS subscale II for parkinsonism	14 (5; 21)	4 (0; 17)	1 (0; 15)	12 (0; 21)
	U = 578.50; *p* = 0.197		U = 5842.50; *p* = *0.019*	
Expressive automatic movements	0 (0; 2)	0 (0; 2)	0 (0; 2)	0 (0; 2)
	U = 770.50; *p* = 0.947		U = 5148.50; *p* = 0.528	
Bradykinesia	1 (0; 3)	0 (0; 2)	0 (0; 1)	1 (0; 2)
	U = 683.00, *p* = 0.511		U = 5484.00; *p* = 0.128	
Rigidity	4 (0; 8)	0 (0; 6)	0 (0; 6)	2 (0; 8)
	U = 619.00, *p* = 0.266		U = 5859.50; *p* = **0.010**	
Gait and posture	1 (0; 2)	0 (0; 2)	0 (0; 1)	1 (0; 2)
	U = 662.00; *p* = 0.419		5520.50; *p* = 0.102	
Tremor/ESRS subscore hyperkinesia	8 (1; 10)	0 (0; 6)	0 (0; 6)	0 (0; 9)
	U = 465.50; *p* = *0.023*		U = 5763.00; *p* = *0.015*	
Postural stability	0 (0; 1)	0 (0; 1)	0 (0; 0)	0 (0; 1)
	U = 807.00; *p* = 0.828		U = 5300.00; *p* = 0.224	
ESRS subscale VI (CGI-S of parkinsonism)	4 (1; 4)	2 (0; 4)	2 (0; 4)	3 (0; 4)
	U = 634.50; *p* = 0.350		U = 554.00; *p* = 0.105	
SRS subscale VIII (stage of parkinsonism)	2 (1; 3)	2 (0; 3)	1 (0; 3)	2 (0; 3)
	652.50; *p* = 0.407		U = 5596.50; *p* = 0.080	
ESRS subscore hypokinesia	7 (0; 14)	0 (0; 12)	0 (0; 11)	4 (0; 13)
	U = 650.50; *p* = 0.394		U = 5597.00; *p* = 0.076	

Data are expressed as median (Q1; Q3) and compared with Mann–Whitney U test. C allele carriers = CC homozygotes + CT heterozygotes; T allele carriers = TT homozygotes + CT heterozygotes. Statistically significant result is shown in bold, whereas nominally significant results, rejected after the Bonferroni correction, are shown in italics.

**Table 4:** ESRS-based AIP scores in haloperidol-treated schizophrenia patients with different *SLC6A3* 3′UTR VNTR alleles.

Parkinsonism Examination Instrument	ESRS-Based AIP Scores in Different *SLC6A3* Allele Carriers			
	10R Homozygotes	9R Carriers	9R Homozygotes	10R Carriers
ESRS subscale II for parkinsonism	2 (0; 15)	8 (0; 20)	1 (0; 20)	5 (0; 17)
	U = 6694.00; *p* = 0.082		U = 1698.50; *p* = 0.750	
Expressive automatic movements	0 (0; 1)	0 (0; 2)	0 (0; 2)	0 (0; 2)
	U = 6139.00; *p* = 0.589		U = 1664.00; *p* = 0.853	
Bradykinesia	0 (0; 1)	0 (0; 2)	0 (0; 1)	0 (0; 2)
	U = 6111.00; *p* = 0.646		U = 1658.50; *p* = 0.876	
Rigidity	0 (0; 6)	0 (0; 8)	0 (0; 4)	0 (0; 6)
	U = 6286.50; *p* = 0.373		U = 1740.50; *p* = 0.592	
Gait and posture	0 (0; 1)	0 (0; 2)	0 (0; 2)	0 (0; 2)
	U = 6244.00; *p* = 0.433		U = 1653.00; *p* = 0.894	
Tremor/ESRS subscore Hyperkinesia	0 (0; 6)	0 (0; 8)	0 (0; 8)	0 (0; 6)
	U = 6617.00; *p* = 0.079		U = 1596.00; *p* = 0.893	
Postural stability	0 (0; 0)	0 (0; 1)	0 (0; 1)	0 (0; 1)
	U = 6153.00; *p* = 0.510		U = 1510.50; *p* = 0.545	
ESRS subscale VI (CGI-S of parkinsonism)	1 (0; 4)	3 (0; 4)	3 (0; 4)	2 (0; 4)
	U = 6994.50; *p* = *0.016*		U = 1521.50; *p* = 0.661	
SRS subscale VIII (Stage of parkinsonism)	1 (0; 3)	2 (0; 3)	2 (0; 3)	2 (0; 3)
	U = 6870.00; *p* = *0.030*		U = 1694.00; *p* = 0.761	
ESRS subscore hypokinesia	0 (0; 11)	0 (0; 13)	0 (0; 10)	0 (0; 12)
	U = 6254.50; *p* = 0.435		U = 1717.50; *p* = 0.680	

Data are expressed as median (Q1; Q3) and compared with Mann–Whitney U test. In addition, 9R carriers = homozygotes for 9 repeats + 9R/10R heterozygotes; 10R carriers = homozygotes for 10 repeats + 9R/10R heterozygotes. Nominally significant results, rejected after the Bonferroni correction, are shown in italics.

**Table 5:** ESRS-based AIP scores in haloperidol-treated schizophrenia patients with different combinations of *HTR6* rs1805054 and *SLC6A3* 3′UTR VNTR alleles.

Parkinsonism Examination Instrument	ESRS-Based AIP Scores in Carriers of Different *HTR6*/*SLC6A3* Allele Combinations		
	*HTR6* T/*SLC6A3* 9R	*HTR6* C/*SLC6A3* 9R or *HTR6* T/*SLC6A3* 10R	*HTR6* C/*SLC6A3* 10R
ESRS subscale II for parkinsonism	12 (0; 21)	10 (0; 19)	0 (0; 10)
	H = 11.44; *p* = **0.003**		
Expressive automatic movements	0 (0; 2)	0 (0; 2)	0 (0; 1)
	H = 1.77; *p* = 0.413		
Bradykinesia	0 (0; 2)	0 (0; 2)	0 (0; 1)
	H = 4.11; *p* = 0.128		
Rigidity	0 (0; 10)	0 (0; 6)	0 (0; 2)
	H = 7.69; *p* = *0.021*		
Gait and posture	0 (0; 2)	0 (0; 2)	0 (0; 1)
	H = 5.17; *p* = 0.075		
Tremor/ESRS subscore hyperkinesia	0 (0; 9)	0 (0; 8)	0 (0; 0)
	H = 12.89; *p* = **0.002**		
Postural stability	0 (0; 1)	0 (0; 1)	0 (0; 0)
	H = 3.45; *p* = 0.179		
ESRS subscale VI (CGI-S of parkinsonism)	3 (0; 4)	3 (0; 4)	0 (0; 3)
	H = 9.68; *p* = **0.008**		
SRS subscale VIII (stage of parkinsonism)	2 (0; 3)	2 (0; 3)	0 (0; 2)
	H = 9.10; *p* = **0.011**		
ESRS subscore hypokinesia	1 (0; 15)	3 (0; 12)	0 (0; 5)
	H = 4.84; *p* = 0.089		

Data are expressed as median (Q1; Q3) and compared with Kruskal–Wallis H test. *HTR6* T/*SLC6A3* 9R = combined carriers of *HTR6* T and *SLC6A3* 9R alleles; *HTR6* C/*SLC6A3* 9R or *HTR6* T/*SLC6A3* 10R = carriers of either HTR6 T or *SLC6A3* 9R allele; *HTR6* C/SLC6A3 10R = combined carriers of *HTR6* C and *SLC6A3* 10R alleles (combined carriers of neither *HTR6* T nor *SLC6A3* 9R alleles). Statistically significant results are shown in bold, whereas the nominally significant result, rejected after Bonferroni, is shown in italics.

## Data Availability

Data available on request.

## Supplementary Materials

The following supporting information can be downloaded at: https://www.mdpi.com/article/10.3390/biomedicines10123237/s1, Table S1: ESRS-based AIP scores in haloperidol-treated schizophrenia patients with different *HTR2C* rs3813929 alleles; Table S2: ESRS-based AIP scores in haloperidol-treated schizophrenia patients with different *HTR2C* rs518147 alleles; Table S3: ESRS-based AIP scores in haloperidol-treated schizophrenia patients with different *HTR2C* rs3813929-rs518147 haplotypes; Table S4: ESRS-based AIP scores in haloperidol-treated schizophrenia patients with different *HTR6* rs1805054 genotypes; Table S5: ESRS-based AIP scores in haloperidol-treated schizophrenia patients with different *SLC6A3* 3′UTR VNTR genotypes.
