# Supplementary material for: SLC6A3, HTR2C and HTR6 Gene Polymorphisms and the Risk of Haloperidol-Induced Parkinsonism
Source: Biomedicines. 2022 Dec 13;10(12):3237. doi: 10.3390/biomedicines10123237 (PMC9776373; doi:10.3390/biomedicines10123237)
Supplement: Supplementary file 1 [file biomedicines-10-03237-s001.zip › biomedicines-2068615-supplementary.pdf]

Supplementary table S1. ESRS based AIP scores in haloperidol-treated schizophrenia patients with different *HTR2C* rs3813929 alleles.

**Parkinsonism examination instrument**	**ESRS based AIP scores in different *HTR2C* rs3813929 allele carriers**	
	C	T
ESRS subscale II for parkinsonism	6 (0; 18)	4 (0; 17)
	U=3731.50: p=0.747	
Expressive automatic movements	0 (0; 2)	0 (0; 1)
	U=3761.00; p=0.794	
Bradykinesia	0 (0; 2)	0 (0; 2)
	U=3785.00; p=0.853	
Rigidity	0 (0; 6)	0 (0; 6)
	U=3719.50, p=0.702	
Gait and posture	0 (0; 2)	0 (0; 2)
	U=3809.00; p=0.907	
Tremor / ESRS subscore Hyperkinesia	0 (0; 8)	0 (0; 6)
	U=3737.50; p=0.731	
Postural stability	0 (0; 1)	0 (0; 0)
	U=3680.00; p=0.563	
ESRS subscale VI (CGI-S of parkinsonism)	2 (0; 4)	3 (0; 4)
	U=4019.00; p=0.637	
SRS subscale VIII (Stage of parkinsonism)	2 (0; 3)	2 (0; 3)
	U=3795.50; p=0.882	
ESRS subscore Hypokinesia	0 (0; 12)	0 (0; 9)
	U=3780.00; p=0.846	

Data are expressed as median (Q1; Q3) and compared with Mann-Whitney U test. Since the *HTR2C* gene is located on the X chromosome and study enrolled only male subjects, for the rs3813929 polymorphism only allele carriers could be analyzed.

Supplementary table S2. ESRS based AIP scores in haloperidol-treated schizophrenia patients with different *HTR2C* rs518147 alleles.

**Parkinsonism examination instrument**	**ESRS based AIP scores in different *HTR2C* rs518147 allele carriers**	
	C	T
ESRS subscale II for parkinsonism	6 (0; 18)	4 (0; 17)
	U=3731.50: p=0.747	
Expressive automatic movements	0 (0; 2)	0 (0; 1)
	U=3761.00; p=0.794	
Bradykinesia	0 (0; 2)	0 (0; 2)
	U=3785.00; p=0.853	
Rigidity	0 (0; 6)	0 (0; 6)
	U=3719.50, p=0.702	
Gait and posture	0 (0; 2)	0 (0; 2)
	U=3809.00; p=0.907	
Tremor / ESRS subscore Hyperkinesia	0 (0; 8)	0 (0; 6)
	U=3737.50; p=0.731	
Postural stability	0 (0; 1)	0 (0; 0)
	U=3680.00; p=0.563	
ESRS subscale VI (CGI-S of parkinsonism)	2 (0; 4)	3 (0; 4)
	U=4019.00; p=0.637	
SRS subscale VIII (Stage of parkinsonism)	2 (0; 3)	2 (0; 3)
	U=3795.50; p=0.882	
ESRS subscore Hypokinesia	0 (0; 12)	0 (0; 9)
	U=3780.00; p=0.846	

Data are expressed as median (Q1; Q3) and compared with Mann-Whitney U test. Since the *HTR2C* gene is located on the X chromosome and study enrolled only male subjects, for the rs518147 polymorphism only allele carriers could be analyzed.

Supplementary table S3. ESRS based AIP scores in haloperidol-treated schizophrenia patients with different *HTR2C* rs3813929-rs518147 haplotypes.

**Parkinsonism examination instrument**	**ESRS based AIP scores in different *HTR2C* rs3813929-rs518147 haplotype carriers**		
	CC	CG	TC
ESRS subscale II for parkinsonism	0 (0; 20)	9 (0; 18)<br>H=1.14; p=0.567	5 (0; 17)
Expressive movements	0 (0; 1)	0 (0; 2)<br>H=0.35; p=0.838	0 (0; 1)
Bradykinesia	0 (0; 1)	0 (0; 2)<br>H=0.97; p=0.617	0 (0; 2)
Rigidity	0 (0; 6)	0 (0; 6)<br>H=0.20; p=0.905	0 (0; 6)
Gait and posture	0 (0; 2)	0 (0; 2)<br>H=0.88; p=0.644	0 (0; 2)
Tremor / ESRS subscore Hyperkinesia	0 (0; 7)	0 (0; 8)<br>H=0.47; p=0.789	0 (0; 6)
Postural stability	0 (0; 0)	0 (0; 1)<br>H=0.56; p=0.755	0 (0; 0)
ESRS subscale VI (CGI-S of parkinsonism)	0 (0; 3)	3 (0; 4)<br>H=4.53; p=0.104	3 (0; 4)
SRS subscale VIII (Stage of parkinsonism)	0 (0; 3)	2 (0; 3)<br>H=2.50; p=0.287	2 (0; 3)
ESRS subscore Hypokinesia	0 (0; 12)	2 (0; 12)<br>H=0.57; p=0.754	3 (0; 9)

Data are expressed as median (Q1; Q3) and compared with Kruskal-Wallis H test.

Supplementary table S4. ESRS based AIP scores in haloperidol-treated schizophrenia patients with different *HTR6* rs1805054 genotypes.

**Parkinsonism examination instrument**	**ESRS based AIP scores in different *HTR6* rs1805054 genotype carriers**		
	CC	CT	TT
ESRS subscale II for parkinsonism	1 (0; 15)	12 (0; 21)<br>H=6.06, *p=0.048*	14 (5; 21)
Expressive automatic movements	0 (0; 2)	0 (0; 2)<br>H=0.41; p=0.816	0 (0; 2)
Bradykinesia	0 (0; 1)	1 (0; 2)<br>H=2.40; p=0.302	1 (0; 3)
Rigidity	0 (0; 2)	2 (0; 8)<br>H=6.90; *p=0.032*	4 (0; 8)
Gait and posture	0 (0; 1)	0 (0; 2)<br>H=2.85; p=0.241	1 (0; 2)
Tremor / ESRS subscore Hyperkinesia	0 (0; 6)	0 (0; 9)<br>H=8.89; **p=0.012**	8 (1; 10)
Postural stability	0 (0; 0)	0 (0; 1)<br>H=1.77; p=0.413	0 (0; 1)
ESRS subscale VI (CGI-S of parkinsonism)	2 (0; 4)	3 (0; 4)<br>H=2.92; p=0.232	4 (1; 4)
SRS subscale VIII (Stage of parkinsonism)	1 (0; 3)	2 (0; 3)<br>H=3.23; p=0.199	2 (1; 3)
ESRS subscore Hypokinesia	0 (0; 11)	3 (0; 13)<br>H=3.33; p=0.189	7 (0; 14)

Data are expressed as median (Q1; Q3) and compared with Kruskal-Wallis H test. Statistically significant result is shown in bold, while nominally significant results, rejected after the Bonferroni correction, are shown in italics.

Supplementary table S5. ESRS based AIP scores in haloperidol-treated schizophrenia patients with different *SLC6A3* 3′UTR VNTR genotypes.

**Parkinsonism examination instrument**	**ESRS based AIP scores in different *SLC6A3* 3′UTR VNTR genotype carriers**		
	10R/10R	9R/10R	9R/9R
ESRS subscale II for parkinsonism	2 (0; 15)	9 (0; 20)<br>H=3.87; p=0.145	1 (0; 20)
Expressive automatic movements	0 (0; 1)	0 (0; 2)<br>H=0.43; p=0.805	0 (0; 2)
Bradykinesia	0 (0; 1)	0 (0; 2)<br>H=0.31; p=0.856	0 (0; 1)
Rigidity	0 (0; 6)	0 (0; 8)<br>H=1.53; p=0.465	0 (0; 4)
Gait and posture	0 (0; 1)	0 (0; 2)<br>H=0.78; p=0.679	0 (0; 2)
Tremor / ESRS subscore Hyperkinesia	0 (0; 6)	0 (0; 8)<br>H=3.28; p=0.194	0 (0; 8)
Postural stability	0 (0; 0)	0 (0; 1)<br>H=0.61; P=0.737	0 (0; 1)
ESRS subscale VI (CGI-S of parkinsonism)	1 (0; 4)	3 (0; 4)<br>H=5.96; p=0.051	3 (0; 4)
SRS subscale VIII (Stage of parkinsonism)	1 (0; 3)	2 (0; 3)<br>H=5.77; p=0.056	2 (0; 3)
ESRS subscore Hypokinesia	0 (0; 11)	0 (0; 13)<br>H=1.09; p=0.580	0 (0; 10)

Data are expressed as median (Q1; Q3) and compared with Kruskal-Wallis H test. 10R/10R=10 repeats homozygotes; 9R/10R=heterozygotes; 9R/9R=9 repeats homozygotes.
